# The role of FSH and TGF-β superfamily in follicle atresia

**DOI:** 10.18632/aging.101391

**Published:** 2018-03-02

**Authors:** Yu-Lan Chu, Ya-Ru Xu, Wan-Xi Yang, Yi Sun

**Affiliations:** 1 College of Life Sciences, Zhejiang University, Hangzhou 310058, China; * Equal contribution

**Keywords:** follicle atresia, apoptosis, ovary, FSH, TGF-β

## Abstract

Most of the mammalian follicles undergo a degenerative process called “follicle atresia”. Apoptosis of granulosa cells is the main characteristic of follicle atresia. Follicle stimulating hormone (FSH) and the transforming growth factor β (TGF-β) superfamily have important regulatory functions in this process. FSH activates protein kinase A and cooperating with insulin receptor substrates, it promotes the PI3K/Akt pathway which weakens apoptosis. Both Smad or non-Smad signaling of the transforming growth factor β superfamily seem to be related to follicle atresia, and the effect of several important family members on follicle atresia is concluded in this article. FSH and TGF-β are likely to mutually influence each other and what we have already known about the possible underlying molecular mechanism is also discussed below.

## Introduction

Folliculogenesis is a process describing the fate of oocytes and their surrounding somatic cells. Follicular development begins with the formation of primordial follicles which contain arrested primary oocytes and a layer of flat granulosa cells. Primordial follicles will be recruited into the growing follicle pool and proceed to further stages of development. Granulosa cells become cuboidal when primordial follicles mature into primary follicles and they turn into multilayers when they enter the stage of secondary follicles [1]. Upon the formation of secondary follicles, outer granulosa cell layers differentiate into theca cells which encircle the inner granulosa layer and produce androgens for subsequent estradiol biosynthesis [2]. When intervals between granulosa cells become larger and merge together to form an antral, follicles get the name of “antral” follicle. The granulosa cells can be further divided into cumulus cells and mural cells. The wall of antral follicles is lined with mural cells while oocytes are linked to the wall by cumulus cells [1,3]. Only very few follicles, in mammalian less than 1%, will be selected and finally ovulate as mature follicles, while others will undergo a process called follicle atresia, which means degeneration of the follicles.

Follicle atresia is an event that happens in all stages of follicles. It is essential for maintaining ovular environmental homeostasis and the abnormity of follicle atresia causes reproductive diseases such as the polycystic ovarian syndrome and premature ovarian failure [4,5]. Antral stage seems to be the decisive point of final fate, as most of follicles undergo atresia at this stage [1]. The main characteristic of follicle atresia is the apoptosis of oocytes, granulosa cells and theca cells [2,6]. Autophagy and necrosis also exist in this process [7], but they are less understood as most studies on follicle atresia focus on apoptosis. Which part of the follicle first encounters atresia depends on species as well as stages of follicles. In porcine ovaries, apoptotic cells first appear in the lining granulosa layer and are followed later by cumulus cells and oocytes [6]. But in bovine ovaries, apoptosis first occurs at oocytes in preantral follicles and at granulosa cells of follicles in later stages of development [2]. When atresia progresses, increasing number of dead cells contribute to decreased volume, detached somatic cells and collapsed antrums of follicles, and eventually the entire follicle is degenerated [8].

The death ligand-receptor system is the most common trigger of granulosa cell apoptosis. Fas/FasL, tumor necrosis factor (TNF) with its receptors and TNF related apoptosis-inducing ligand (TRAIL) with its receptors all participate in granulosa cell apoptosis in many species [6]. The deficiency of extracellular survival factors in the environment is likely to induce the intrinsic pathway of apoptosis. This involves the release of cytochrome c from mitochondria controlled by the highly conserved bcl protein family [9]. The Bcl-2 family can be divided to two functional classes. One class including Bax, Bak promotes apoptosis, and the other class, including Bcl2, Bcl-xl, has the opposite effect mainly through forming heterodimers with the former class [9]. During follicle atresia in bovine and rat follicles, Bax level is elevated while Bcl-2 level does not change significantly [10]. In porcine ovarian follicles undergoing atresia, expression level of the anti-apoptotic bcl-2 family protein increases together with that of pro-apoptotic genes, possibly to prevent cell death [11]. However, the ratio of them gets out of balance, with relatively higher pro-apoptotic gene expression in atretic follicles at different stages [12]. This indicates that in ovaries, intercellular survival factors can play an essential role in follicle maintenance since intrinsic apoptotic pathways are often related to the lack of survival factors.

The ovular environment is quite complex, with circulating gonadotropins, multiple paracrine and autocrine factors. In early stages of folliculogenesis, it is acknowledged that follicles can develop independently of gonadotropins while ovary derived paracrine factors, like transforming growth factors may have more dominant roles in this process [1]. Interaction between oocytes and granulosa cells is critical to growth and differentiation of follicles. This kind of communication is carried out by intra-ovarian factors and is mutually beneficial [13]. Although the beginning of folliculogenesis is commonly accepted to be “gonadatropin independent”, receptors of gonadotropins are also expressed in follicles before antrum formation and gonadotropins do faciliate folliculogenesis in early stages of follicles [14]. As follicles progress into the antral stage, gonadotropins, especially follicle stimulating hormone, are crucial for follicle survival and growth [14].

The communication between oocytes and somatic cells through cooperation of multiple factors renders delicate regulation of folliculogenesis, steroidogenesis, and resultant normal ovarian functions. However, on the other hand, it also brings greater challenges for researchers to elucidate the molecular mechanisms underlying the sophisticated regulating network. In this article, we comprehensively summarize functions of follicle stimulating hormone and transforming growth factor beta superfamily in controlling follicle atresia. It is the first review to discuss the crosstalk between cAMP/PKA and PI3K/Akt pathways stimulated by follicle stimulating hormone in the ovarian environment and some aspects of the possible molecular mechanism how follicle stimulating hormone and transforming growth factor beta superfamily interact with each other are also suggested here.

### 1. Follicle stimulating hormone

Follicle stimulating hormone (FSH), secreted by pituitary gland, is one of the gonadotropins that belong to the glycoprotein hormone (GPH) family. FSH is a heterodimer consisting of one α and one β subunit, which exhibits pseudo 2-fold symmetry [15]. FSH receptor is a number of G-protein coupled receptors and it belongs to the leucine-rich-repeat-containing G-protein coupled receptor subfamily (LGR) which means it contains the Leucine-rich ectodomain [15]. Like other G-protein coupled receptors, FSH receptors have seven transmembrane helices and transduce signals to downstream molecules through the disassociated Gα subunit of the heterotrimeric G-protein [15]. FSH affects follicular growth, maturation, dominant follicle selection as well as estradiol production [16]. It is considered to be an important survival factor for follicles in the course of folliculogenesis. FSH dampens apoptosis of cultured granulosa cells *in vitro* and protects follicles from atresia *in vivo* [17,18]. The scope of its function is very wide: it can inhibit atresia in follicles of different maturity including antral follicles, preovulatory follicles and dominant follicles and also in too many species to be listed here [19–21]. The inhibition of FSH by octapeptide or FRBI-8 induces atresia and damages ovarian functions [22,23]. Also, the deficiency in FSH receptors contributes to the loss of follicles [24]. FSH reduces the level of FasL, but not Fas, in granulosa cells to interfere Fas/FasL mediated extrinsic apoptosis [25]. The intrinsic apoptotic pathway seems to be more predominantly downregulated by FSH than the extrinsic pathway since molecules related to the former are main factors that respond sensitively to FSH treatment [18]. Many researchers have reported that the cAMP/PKA pathway and PI3K/Akt pathway occupy a significant position in the functions of FSH. In this part, we mainly focus on signaling mechanisms of FSH and depict a network of those two pathways which enables the role of FSH in follicle atresia.

### 2. PKA: the first kinase to be induced by FSH

Once FSH GPCRs is switched on by the formation of GTP from GDP, the Gα subunit disassociates and activates nearby effector enzymes, which generates second messenger to transduce the signal to downstream. In granulosa cells, cAMP is increased by the addition of FSH by over 10 folds [20]. The effect of FSH to suppress apoptotic DNA fragmentation in granulosa cells can also be mimicked by analogs of cAMP [20]. Protein kinase A is in response to cAMP. Importance of this kinase in ovarian functions again illustrates its irreplaceable position in cells and organisms. It appears in multiple processes including granulosa cells differentiation, apoptosis and oocyte maturation. Time-dependent increase of PKA activities stimulated by FSH provides direct evidence that FSH can signal through PKA [26]. The addition of H89, an inhibitor of PKA, blocks the anti-apoptotic effect of FSH in granulosa cells and also neutralizes H3 phosphorylation effect of FSH which leads to granulosa cell differentiation [27,28]. FSH also elevates A-kinase anchoring protein, which may translocate typeIIα PKA to where it is required to phosphorylate substrates [29]. PKA is considered to be the main kinase activated by FSH and cAMP/ PKA pathway can also exert its effects by interacting with other signaling pathways such as PI3K/AKT, which is detailed below. There are some exceptions. For example, in the hGL5 cell line FSH seems to signal independently of cAMP / PKA pathway. Instead, in this case β-arrestins activates the ERK pathway [30]. However, on the whole, different pathways seem to “branch out” from PKA, as is reviewed by Mary Hunzicker-Dunn [16].

### 2.1. FSH mediated by PI3K/AKT

Phosphatidylinositol-3-kinase (PI3K) can be triggered by multiple extracellular signals and then activates serine/threonine protein kinase Akt (PKB). This activation occurs in nearly all types of cells, tissues and organs based on mouse and human genetic studies, and in mammalian ovaries it is crucial for oogenesis as well as folliculogenesis [31,32]. PI3K, a membrane kinase, converts PIP_2_ to PIP_3_. Akt and phosphoinositide-dependent kinase (PDK) each has a pleckstrin homology (PH) domain and this domain enables these two proteins to be recruited by PIP_3_. Then Akt can be phosphorylated by PDK and mammalian target of rapamycin complex (mTORC) on Tyr^308^ and Ser^473^, respectively, and is thus activated to execute its functions [31,32].

Evidence shows that PI3K/Akt pathway is related to apoptosis in ovaries. BMP-7 attenuates granulosa cells apoptosis in cows and heifers by viture of PI3K/PDK-1/Akt pathway. Inhibitors of these three proteins eliminate the effect of BMP-7 and causes apoptosis [33]. Cyclophosphamide is a common chemotherapeutic agent in cancer therapy, but it can lead to a loss of primordial follicles and infertility. In female mice exposed to cyclophosphamide, phos-Akt, phos-mTOR as well as downstream proteins increase, which indicates the activation of PI3K/Akt/mTOR pathway [34]. In 3-Methylcholanthrene treated neonatal ovaries, Bad, one proapoptotic factor in the Bcl-2 family, is phosphorylated by Akt and loses its effects on primordial follicles [35]. This offers us one aspect of how Akt can interfere with the apoptotic pathway. Transgenic mice with constitutive PI3K activity have more primordial follicles with less DNA breakage and Bax expression [36]. These results tell that PI3K/Akt pathway can also reduce the level of functional Bcl-2 factors to prevent atresia in follicles.

Akt has been reported to prevent apoptosis by phosphorylating transcriptional factors including the Forkhead family of transcription factors (FoxOs) which regulate expression of multiple apoptosis related factors [37]. FoxOs are active participants in programmed cell death controlling. They directly upregulate the expression of death receptor ligands including FasL and TRAIL and proapoptotic BH3-only group proteins in Bcl-2 family members to promote apoptosis [38].When not phosphorylated, FoxOs enter the nucleus and work as activators or suppressors. But once they are modified with the phosphate groups, they bind to 14-3-3 proteins in the cytoplasm and are no longer located in the nucleus, thereby losing their original functions [39,40]. So Akt inactivates FoxOs and elminates their proapoptotic role by phosphorylating them and relocating them into the cytoplasm. In mammals, FoxO family has four members: FoxO1, FoxO3, FoxO4 and FoxO6 [41]. With high protein homology, they regulate similar sets of genes with large overlap by binding to a consensus sequence and their specificity may stem from different coregulators [41]. In mammalian ovaries, FoxO1 and FoxO3 both have high expression and their function in follicle atresia is regulated by PI3K/Akt pathway [42]. During oxidative stress introduced apoptosis in granulosa cells, the expression of FoxO1 is upregulated both *in vivo* and *in vitro* [37]. Pro-apoptotic genes elevated by FoxO1 including Bim, FasL, and caspase-3 failed to increase when PI3K/Akt pathway is turned on [37]. FoxO3 level also rises in follicles undergoing atresia. The loss of Rictor subunit in mTORC2 reduces activated Akt and inhibits FoxO3, raising the protein expression of Bad, Bax, and cleaved PARP which leads to accelerated follicle atresia [43]. Bcl2 as well as FasL will be rasied by FoxO3, which indicates that they may be transcriptional targets of FoxO3 [44].

Functions of FSH can be mediated by PI3K/Akt. FSH suppresses expression of Bim_EL_, which induces granulosa cell apoptosis, via the PI3K/Akt pathway [45]. FSH can downregulate Bim_EL_ and reduces apoptosis, but both knockdown of Akt and inhibition of PI3K eliminate this effect. In the presence of FSH, if Akt/PI3K pathway is blocked, FoxO3a is still activated and binds to promoters of Bim_EL_ [45]. These results indicate that PI3K/Akt/FoxO axis may exist as the downstream of FSH. Other FoxO members can also participate in this axis. Inhibition of PI3K/Akt contributes to the translocation of FoxO1 from cytoplasm to nucleus and neutralizes the protective effect of FSH to H_2_O_2_-exposed granulosa cells [46]. If FoxO1 is mutated at Akt phosphorylation sites, FSH loses its anti-apoptosis effect, which further confirms the underlying mechanism of FSH. Intriguingly, in this case, FoxO1 can not only bind to the promoter of Bim, but also binds to the promoter of itself, which forms a positive feedback loop [46].

### 2.2. PKA/PI3K/AKT axis in follicle atresia

PI3K/Akt is the downstream of receptor tyrosine kinases (RTK), which is typically activated by insulin and the insulin like growth factor. Upon the binding of these ligands, RTK undergoes autophosphorylation and opens the activated site to modify the target protein, insulin receptor substrate (IRS) by adding phosphates to its Tyrosine. This modification creates the binding site of PI3K and other proteins containing the SH2 domain such as the adaptor growth factor receptor bound protein 2 (GRB2). The binding of SH2 domains within PI3K to IRS leads to a downstream transduction as described in 3.1. (reviewed in [47]).

In the presence of FSH, PKA seems to stand above PI3K/Akt pathway. M. Shen examined the role of the PKA, PI3K/Akt pathway in inhibiting FoxO1 in dominant follicles treated with FSH [46]. Inhibition of both pathways abrogates the suppressive effect of FSH in H_2_O_2_ induced apoptosis, and inhibition of PKA blocks the function of PI3K/Akt pathway, which shows the probable involvement of the PKA/PI3K/Akt axis in mediating FSH [46]. A study done by Mary E. Hunzicker-Dunn also shows the inhibition of PKA offsets the phosphorylation of Akt induced by FSH [48].

IRS is shown to stand at the point of intersection of PKA and the PI3K/Akt pathway. FSH facilitates both IRS-2 expression and post-transcriptional stability in a cAMP dependent way [49]. FSH can promote the translocation of SP1 to the nucleus and SP1 will bind to the IRS-2 promotor to elevate the level of the IRS-2 [49]. Without IRS-2 the PI3K/Akt pathway can’t be induced by FSH [49]. So, these results together indicate that IRS-2 is the key to link the cAMP/PKA pathway with the PI3K/Akt pathway. In 2012, a model explaining this crosstalk which involves GAB2 and IRS-2 was proposed [48]. In granulosa cells, the adaptor growth factor receptor bound protein 2-associated binding protein 2 (GAB2) acts as the type I regulatory subunit (RI) A-kinase anchoring protein (AKAP) which binds to RI of PKA and in this case forms a complex together with IRS-1 and p85 R-subunit of PI3K [48]. In the presence of FSH, PKA directly phosphorylates GAB2 on Ser^159^. It somehow renders dephosphorylation on Tyr^452^ in GAB2 and this seems to enhance PI3K/Akt signaling. It also phosphorylates Tyr^989^ in IRS-1 in some way, which is proposed to result in the detachment of PI3K from GAB2 and the binding of PI3K to IRS-1 [48]. In 2016, the role of PKA in p-YXXM motif in IRS was further investigated. PKA can activate protein phosphatase 1 (PP1) to directly dephosphorylate inhibitory Ser/Tyr sites and this dephosphorylation can increase the sensibility of IRS to be phosphorylated at Tyr^989^ by the receptors [50]. This result further details the model in 2012.

Insulin like growth factor (IGF) is an important paracrine factor in ovaries affecting follicle development. IGF and IGF binding protein influence atresia in many different species. They are critical to the functions of FSH and they may work synergistically with FSH to affect steroidogenesis and follicle development [51]. In mouse, rats and human granulosa cells, IGF-1 and IGF-1 receptors are essential for FSH to activate Akt [51]. In the axis of PKA/PI3K/Akt, IGF-1 is required to initiate IGF-1R but it is not enough for activation of IRS. The dephosphorylation of inhibitory site on IRS by PP1 is required to achieve this activation [50].

**Figure 1:**
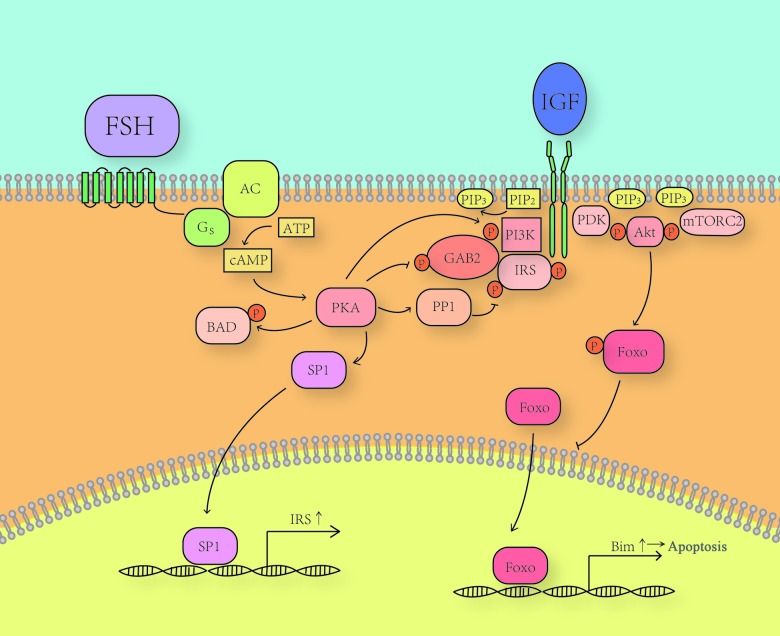
**The model of FSH/PKA/PI3K pathway.** FSH induced PKA activates PP1 to dephosphorylate inhibitory sites on IRS, which facilitates phosphorylation of Tyr^989^ on IRS. Phosphorylated Tyr^989^ leads to the detachment of PI3K from GAB2 and its binding to IRS. PKA directly phosphorylates GAB2 on Ser^159^ and dephosphorylates p-Tyr^452^ through an unknown mechanism. These modifications also promote a rearrangement of the complex and the activation of PI3K. Activated PI3K converts PIP_2_ to PIP_3_, which recruits PDK, mTORC2 and Akt. PDK and mTORC2 together activate Akt. Akt then phosphorylates FoxO and keeps it out of the nucleus and thus abrogates its functions of promoting the expression of some pro-apoptotic genes, such as Bim. PKA can promote the expression of IRS through promoting the translocation of SP1, which interacts with the IRS promotor. PKA also directly phosphorylates Bad and inhibits its function.

The model of FSH/PKA/PI3K pathway is concluded in Figure 1.

### 3. The transforming growth factor β superfamily

The transforming growth factor β superfamily (TGF-β) can be further divided into subfamilies including TGF-β, activin/inhibin, nodal-related proteins, bone morphogenetic protein (BMP), growth and differentiation factor (GDF), glial cell-derived neurotrophic factor (GDNF), and Müllerian inhibitory factor (MIF) according to sequence similarity and signaling specificity. Extensive experiments have revealed its nonnegligible role in follicle development. It regulates cellular functions including proliferation, apoptosis, differentiation, steroidogenesis, and cell specification via paracrine and autocrine pathways. Activated TGF-β ligands are usually dimers covalently linked by one intermolecular disulfide bond and feature a “cysteine knot” structural motif which consists of three intramolecular disulfide bonds [52]. After binding to cell surface receptors which belong to the receptor serine/ threonine kinase family, they induce TGF-β/smad signaling pathway that modulates activation or repression of the expression of several hundred genes [53]. TGF-β family members are important factors in the reproduction system [54]. Among those members, BMP-15, GDF-9 and activin/inhibin close relate to regulation of follicle atresia and their functions in this process will be detailed below.

### 3.1. The signaling mechanism of TGF-β superfamily

TGF-β superfamily binds to the cell surface by TGF-β type I and II receptors, which are classified as serine-threonine kinases [55]. Both TGF-β type I and II receptors contain one intracellular serine/threonine kinase domain, one single-pass transmembrane region, and one extracellular TGF ligand binding domain. The TGF-β type I receptor has the characteristic GS (glycine/serine rich) domain, which is named after a TTSGSGSG sequence at its core [56]. Smad proteins including R-smad, Co-smad and I-smad are downstream molecules for intracellular TGF-β signalling. Upon the binding of mature TGF-β ligands in the form of homodimers or heterodimers, two type I receptors and two type II receptors are brought together to form a stable heterotetramer, although there may be some differences between TGF-β/activin and BMP/GDF in terms of how they bind and assemble their receptors [55]. TGF-β type II receptors then phosphorylate TGF-β type I receptors at their GS domains and activated TGF-β type I receptors then phosphorylate two serine sites in R-smad at their C-terminal SXS motif [57]. The downstream R-smads show specificity with different ligands as they bind to different receptor heterotetramers. The activation of type I receptor ALK4/5/7 by TGF-β/Activin/Nodal leads to the phosphorylation of smad2/3 while the phosphorylation of ALK1/2/3/6 activates smad1/5/8 [56]. The conformational changes of R-smad induced by phosphorylation allow two R-smad to combine with one Co-smad (smad-4) to form a trimer which can come into the nucleus together with other transcriptional factors, to activate or suppress downstream genes [56]. Smad6 and smad7 are I-smads that inhibit TGF-β signaling in many aspects including the ub-mediated degradation, dephosphorylation and complex interference [58]. There are also situations where TGF-β signals through non-smad pathways, such as MAPK pathway and PI3K/AKT pathway [59].

TGF-β signaling pathway takes part in multiple aspects in ovarian functions and is of great importance in the course of egg maturation in ovaries. Smad5 is related to the Fas/FasL apoptosis pathway in follicles [60]. Depletion of Co-smad smad4 in mouse ovaries causes endocrine disorders and increased follicle atresia [61]. Accordingly, when I-smad smad7 is overexpressed, the rate of apoptosis increased dramatically [62]. These results indicate that the TGF-β signaling pathway participates in folliculogenesis and may influence follicle atresia. Also, the Non-smad pathway of TGF-β signaling is likely to function in follicle atresia, as TGF-β1 induced ERK/Akt can promote survival and the FoxO transcriptional factor is also involved in the TGF-β1 stimulated PI3K.Akt downstream [63,64].

### 3.2. BMP-15

The bone morphogenetic protein family (BMP) was first known for its function in cartilage and bone formation at an extraskeletal ectopic site, and subsequent studies have unveiled its influential role in bone homeostasis and its regulation of bone development and repair [65]. But its effects reach far beyond what its name indicates. Other than impacts on bone metabolism, it is also related to functions of cardiovascular organs, as well as the reproductive and nervous system [66]. BMP-15 (also GDF-9B) is an exception in the TGF-β family in terms of its structure. It lacks the conserved cysteine that forms the disulphide with others while most of other members have that structure [67]. BMP-15 signals through type II receptor BMPR2, which in this case interacts mainly with type I receptor BMPR1B (also called ALK-6), and triggers Smad1/5/8 as downstream molecules [68,69].

Data from studies on several different species including goat, swine, cow, and mouse show the involvement of BMP in preantral follicle activation and maintenance, culumus expansion and stabilization, ovulation and embryonic development, as well as corpus luteum activity [70–72]. Both BMP-15 and its two types of receptors, BMPR1B and BMPR2 have been reported to be expressed in theca and granulosa cells of follicles at different stages including preantral follicles (including primordial, primary and secondary follicles) and antral follicles [72–75].

In the antral stage, BMP-15 prevents cumulus cells from apoptosis [76,77]. It elevates the expression of the anti-apoptotic factor Bcl2 while suppressing that of pro-apoptotic factor Bax in bovine ovaries, showing its pro-survival effect [76]. Based on ultrastructural analysis, treatment of BMP-15 helped to maintain the ultrastructural integrity of the preantral follicles, while follicles cultivated without BMP-15 exhibited some signs of cellular stress in the course of observation [73,75]. The Chemokine, C-C motif ligand 2 (CCL2) is a gene that regulates cell death in primary T-cells and cardiac myocytes, and it delays the apoptosis of neutrophils by activation of PI3K/Akt and the NF-kB pathway [78,79]. Bo Zhai’s work showed that BMP-15 may prevent the apoptosis of cumulus cells in cooperation with CCL2 [77]. Kit ligand, also called stem cell factor, is known to promote granulosa cell proliferation, and can function as an anti-apoptotic factor of follicle growth at different stages [80,81]. BMP-15 can stimulate KL expression in mammalian ovaries, and it seems that there exists a negative feedback loop between BMP-15 and KL while BMP-15 directly elevates the level of KL. KL can negatively affect the expression of BMP-15 [82]. However, a study carried out by Mark A. Fenwick indicates a negative role of BMP-15 in preantral follicle growth in mice. Preantral follicles exposed to BMP-15 were observed a significant increase in follicle size in the first day, but as time went on, follicles cultivated by BMP-15 showed the appearance of shrinkage and an increase in apoptotic activities of granulosa cells [83].

The expression of BMP receptors (BMPR1A, BMPR1B and BMPR2) are elevated in histologically atretic follicles while BMP-15 mRNA levels decrease [68]. Bernardo’s study shows that the BMPR1B mRNA expression increases in subordinate follicles at the time of deviation and follicles treated with pro-apoptosis agents. These correspond to previous studies, where the regulation of BMPR by estradiol is tested [69]. When the estradiol level is lowered, BMPR1B mRNA expression is elevated, which is just as the situation in subordinate follicles compared to healthy follicles [69]. Probably, high BMPR1B mRNA expression is a sign indicating us that dominant follicles are in an unfavorable condition. Also, there is a possibility that the increase in BMP receptors is a compensation for low level of BMP in the follicular environment. Influences of BMP-15 on follicle development may thus be stage-specific and there may also be some differences between subjects of polyovaries and of monoovaries. What should be noted is that the concentration of BMP-15 used in these studies may affect the authenticity of the results *in vitro*, as a higher concentration of BMP-15 may be required to simulate the ovarian environment.

### 3.3. GDF-9

GDF-9 is an oocyte-derived factor and it is the first one among other oocyte-secretion factors that is indicated to influence somatic cells. It activates Smad2/3 transducers through typeII receptor BMPR2 and type 1 activin receptor-like kinase receptor 5 (ALK5). It was first discovered in 1993 and another study showed that unlike GDF-3, its expression mainly appeared in oocyte [84] although later studies detected it in non-ovarian tissues such as testis, pituitary, hypothalamus, uterus, and bone marrow [84,85]. Study conducted on GDF-9 deficient mice showed that GDF-9 was vital to follicle development as the lack of GDF-9 leads to the blockage of growth after the primary stage [86]. In many species, GDF-9 is found to express first after the primordial stage. However, in some species, it is likely to initiate the formation of pregranulosa cells by regulating the differentiation of somatic cells and thus promoting the formation of primordial follicles [87]. GDF-9 leads primary and secondary follicles to later stages and it stimulates the production of inhibin-A, which is a marker for differentiation in early follicles [88,89]. GDF-9 can increase the number of theca cells. It is indicated that the primary target of GDF-9 are theca cells rather than granulosa cells as theca cells have a higher sensibility to GDF-9 [90].

Insulin-like 3 (INSL3) is a paracrine factor that mainly expresses in theca cells which stimulates preantral follicle growth and INSL3 deficient mice exhibit a high rate of follicle atresia and luteolysis [91]. Its function may be mediated by GDF-9 via the cAMP signaling pathway [92]. In caprine preantral follicles cultivated with FSH and Thyroid Hormone, the inhibitory of GDF-9 induces a higher rate of apoptosis and attenuates the growth of the follicles [93]. GDF-9 is likely to be an antiapoptotic factor during the transition from preantral to early antral follicles according to the research carried out by Makoto Orisaka in 2006 [94]. The injection of GDF-9 Morpholino antisense oligos led to a rise in caspase-3 activities and induced follicle atresia in bovine preantral follicles [94]. There is also a study conducted in prostate cancer cell lines, in which GDF-9 acts as a survival factor for cancer cells and suppresses caspase-3 dependent apoptosis. These results indicate the involvement of GDF-9 in cell apoptosis by affecting caspase activities. In porcine cumulus cells, GDF-9 suppresses the incidence of follicle atresia [95]. The level of Bcl-2-interacting mediator of cell death-extra long (BIM_EL_), which is a proapoptotic factor, is lowered by GDF-9, and this may be accomplished by the stimulation of the PI3K/FoxO3a pathway [95]. This result is in accordance with a previous study that GDF-9 had a protective effect on ceramide-induced apoptosis in granulosa cells. But this effect was attenuated by the blockage of the PI3K/Akt pathway [94]. Preantral follicles exposed to GDF-9 were observed a long-term promotion in follicle growth, and the negative effects of BMP-15 on preantral follicles after a short period of cultivation is counteracted by GDF-9, and this suggests on cooperative effects of GDF-9 and BMP-15 in ovaries [83]. GDF-9 and BMP-15 are two closely related paralogs in ovaries, and GDF-9 shares the special structure of lacking one cysteine with BMP-15. They can both function in the form of monomers and homodimers, but recent studies have also emphasized the role of GDF-9:BMP-15 heterodimers in ovular functions [93,96]. How different forms of these two oocyte-derived factors affect follicle atresia and how they interact in their regulation of ovular dynamics still require further study.

### 3.4 Activin/Inhibin

Activin was originally discovered in the 1980’s and was named for its activating efficacy in releasing FSH from pituitary gonadotropines [97]. Activins are homogenous or heterogenous dimers consisting of four already known subunits, βA, βB, βC and βE. They are designated according to their subunits, for example, Activin A contains two βA groups, Activin AB contains one βA and one βB [98]. While Acitivin A seems to be the most potent and well-studied regulator of ovaries compared with other kinds of Activins, few researches have reported the expression of βC and βE in ovaries. Activin mediates proliferation and differentiation in ovaries through type I receptor ALK-4 and type II receptor ActR2/2B and requires smad2/3 as mediators [66]. Inhibin was first discovered in testis by Mottram and Cramer in the 1920’s, and its possible role in regulating pituitary function was also described [99]. And in the 1980’s, it was isolated from follicular fluids and in this elution process, Activin was also first isolated [99]. Further study reveals that Sertoli cells in testis and granulosa cells in ovaries are the main producers of Inhibin [100]. Inhibin is reported to have antagonistic effects with Activin in many aspects since it can block the binding of Activin to its receptors through competition thereby cutting off the downstream pathway [101].

There is one α-subunit and two β-subunits, that is βA-subunit and βB-subunit. α-subunits in different types of inhibins are the same, while inhibin A has one βA-subunit and inhibin B has one βB-subunit [98]. α-subunit and β-subunit form a dimer linked by disulphide bonds. Inhibin is shown to have an antagonistic effect on follicle atresia. When cultivated with Inhibin A, apoptosis in human granulosa cells is suppressed with a rise in the level of anti-apoptosis factor Bcl-2, Bcl-xl, and a reduction in the expression of proapoptotic factors Bax and Caspase-3 [102]. Accordingly, knockdown of inhibin A leads to a higher incidence of apoptosis in gruanulosa cells [103]. In transgenic mice whose inhibin-α subunits are interfered, the number of atretic follicles increases and the progression of cell proliferation is impeded [104]. Silencing of inhibin βB in primary mouse granulosa cells also shows negative effects on follicle development as this interference increases apoptosis and arrest during the G1 phase [105]. This indicates that inhibin is required for normal development of follicles and without the effect of inhibin, the balance between survival and death may be broken down.

Activin has a stimulatory effect on the survival of oocytes and the formation of primordial follicles via smad2/smad3 signaling and this may relate to the function of the kit ligand system [106,107]. Oocyte-somatic interaction is maintained by Activin in preantral follicles thus the integrity of follicles is associated with the function of Activin [108]. Activin also fosters granulosa cell proliferation, preantral follicle growth as well as antral formation [108,109]. When treated with Activin, the proportion of atretic follicles is reduced in cultured rat ovaries [110]. And the decrease in caspase-6 indicates its suppressive effect on apoptosis [109]. Activin alone facilitates the survival of preantral follicles both *in vitro* and in a 3-dimentional environment and the addition of fibroblasts seems to enhance this function [111,112]. These results indicate a positive role of Activin in folliculogenesis.

### 4. Interaction between TGF-β and FSH signaling

Activin and Inhibin, as members in TGF-β superfamily, are well known to affect the releasing of FSH from gonadotrope cells, as reviewed by Daniel J. Bernard [60]. Actually, in granulosa cells, TGF-β superfamily can affect functions of FSH by influencing FSH receptors. Studies have shown that TGF-β superfamily members including GDF9, Activin, and several BMPs, have the ability to stimulate the expression of FSHR, and can increase the stability of FSHR mRNA in granulosa cells [113–115]. This effect can be strengthened by FOXL2 factor, which can directly interact with Smad, the downstream molecules of TGF-β signaling [113]. When Smad is absent, FSH loses its functions in ovulation and follicle development both *in vitro* and *in vivo*, which indicates that Smad may affect FSH function in some way [113]. Smad is confirmed to bind to Smad binding element (SBE) in FSHR promotors [113] and FOXL2 may work as a partner of Smad in the forkhead-binding element (FBE) near SBE [116].

Intriguingly, FSH in turn affects the expression and function of both TGF-β family members as well as their receptors. Evidence shows that FSH promotes the expression of TGF-β and activin [117]. As for the level of TGF-β receptors, some report a decrease of TGF-βRI, TGF-βRII, BMPRII and ALK-5 induced by FSH alone [117,118]. However, when treated with estradial together, FSH can dramatically promote their expression [118]. The ovary is a complex environment, and the treatment with both FSH and estradial may be closer to the genuine situation, which means FSH has an overall positive effect on the expression of TGF-β receptors. This may be explained by AS160 phosphorylation induced by FSH. AS160, which is a guanosine triphosphatase (GTPase)-activating protein, is typically known for its role in vesicular traffic of the glucose transporter GLUT4. As is reviewed by Kei Sakamoto, Akt phosphorylates AS160, mainly at Thr^642^ and inactivates it, which fosters the formation of Rab GTPase to help the fusion of GLUT4 loaded vesicular to the membrane [119]. One recent research shows that TGF-β receptors can also be the cargo whose translocation to the membrane is promoted by Akt phosphorylated AS160 [120]. In the ovary, insulin and insulin like growth factor can both initiate PI3K/Akt pathway and as discussed in part 2, PKA stimulated by FSH may be essential in enabling Akt to execute its functions related to granulosa cell survival. These together indicate a possible mechanism of FSH to participate in translocating transmembrane TGF-β receptors to the cell membrane and thus enabling the normal function of TGF-β factors.

TGF-β factors have been reported to facilitate FSH functions in gap junction formation and steroidogenesis by enhancing the activity of CREB-regulated transcription coactivator (CRTC2, also known as TORC2) [121,122]. TORC2 can sense both cAMP/PKA and calcium signaling induced by FSH [122]. In basal condition, phosphorylated TORC2 is bound to 14-3-3 proteins and locates in the cytoplasm. However, when these two signalings are induced by FSH in granunolsa cells, calcineurin dephosphorylates it meanwhile cAMP/PKA protects it from phosphorylation by other kinases, which may be SIK2 [122,123]. Then, TORC2 can translocate into the nuclues and strengthen the activity of cAMP-response element binding protein (CREB), which is a transcriptional activator response to FSH/cAMP/PKA. CREB-Bcl2 signaling has been shown to be a survival signaling in different cell lines [124]. Other than raising Bcl2 level, CREB decreases Bim expression, diminishes phosphorylation of Bim and maintains the association between the microtubule network, thereby reducing apoptosis [125]. TGF-β1 can act through TGF-βR1 to facilitate the function of FSH when it is treated with FSH by enhancing TORC2 activities. FSH’s stimulation of TORC2 may not be a lasting effect. A later rise as well as translocation of TORC2 requires synergia of TFGβ1 [121,122]. And in this process, rather than PKA, calcineurin seems to matter, which requires further study to figure out the molecular mechanism underlying TGF-β affected calcineurin activities. More than enhancing CREB functions, TORC2 is also known for the activation of Akt, as mentioned above. TORC2 is also required for the phosphorylation and activation of Akt. The inhibition of TORC2 attuenes Akt stimulation and induces apoptosis [126]. This indicates us that TGF-β factors may also enhance FSH induced PI3K/Akt pathway via enhancing mediator TORC2.

TGF-β can enhance FSH stimulated progesterone production and luteinizing hormone (LH) receptor expression [127,128]. Both progesterone and LH can promote the survival of preovulatory follicles. In rat preovulatory follicles, luteinizing hormone (LH) elevates the level of IGF-1 and suppresses follicular apoptosis [129]. Progesterone suppresses apoptosis in rat and human granulosa cells through both nuclear and membrane receptors [130,131]. Additional treatment of TGF-β in the presence of FSH can elevates the expression of steroidogenic acute regulatory (StAR) protein, 3b-hydroxysteroid dehydrogenase (3bHSD) and cholesterol side-chain cleavage enzyme (P450scc), which are important proteins in progesterone production [127]. It is likely that TGF-β and FSH can promote follicle survival indirectly through increasing the concentration of progesterone and enhancing the responsiveness to LH.

**Figure 2:**
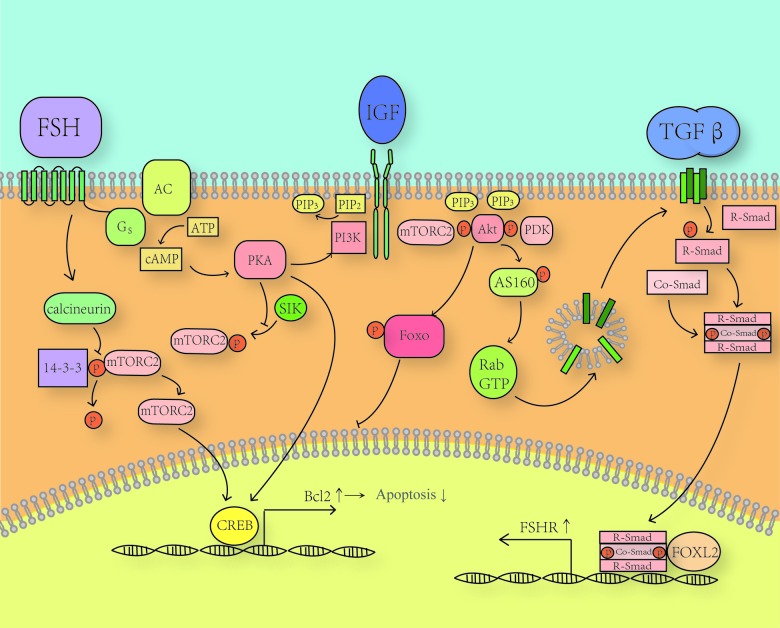
**Crosstalk between TGF-β signaling and PKA/PI3K/Akt axis.** TGF-β signals through tetramer receptors as dimers. TGF-β receptors phosphorylate R-Smad and two phosphorylated R-Smad forms a complex with one Co-Smad. This complex can enter the nucleus and bind to the Smad-binding element in the FSHR promotor and promote the expression of FSH receptors. FOXL2 can strengthen this effect by binding to a forkhead-binding element near the Smad-binding element. Akt induced by FSH through PKA can phosphorylate AS160 mainly at Thr^642^, which is a guanosine triphosphatase (GTPase)-activating protein. Then activated GTPase proteins facilitate the fusion of TGF-βR loaded vesicles to the membrane.

Figure 2 concludes the network of how FSH and TGF-β factors affect each other in a beneficial way, which again reminds us of complex ovular environment and the necessity to consider events happening in the ovary as the result of overall effect of multiple factors.

## CONCLUSION

Follicle atresia has important physiological functions in the female mammal reproductive system. It is based on cell apoptosis and is influenced by multiple factors in the ovary. FSH may be the most critical pro-survival factor for follicles. It functions through integrated signaling pathways where PKA is the major transducer. The crosstalk between different pathways is common and complex, and to follicle survival, interaction between PKA mad PI3K pathways is likely indispensable. Among various intra-ovarian paracrine and autocrine factors, the TGF-β superfamily is best studied in terms of its effect on follicle atresia. The cooperation and mural effects of FSH and TGF indicate the delicate management of the follicles. Elucidation of the molecular mechanism underneath follicle atresia may provide new insights into disorders related to intensified atresia and further the development of clinical treatments.
